# Efficacy and Safety of Rezafungin Versus Caspofungin for the Treatment of Candidemia and Invasive Candidiasis in a China Cohort of a Double‐Blind, Randomised, Phase 3 Trial (ReSTORE China)

**DOI:** 10.1111/myc.70122

**Published:** 2025-11-05

**Authors:** Haihui Huang, Sizhou Feng, Yunsong Yu, Yong Zhang, Yuan Yuan, Laura Cox, Yingyuan Zhang

**Affiliations:** ^1^ Institute of Antibiotics, Huashan Hospital, Fudan University Shanghai China; ^2^ Institute of Hematology and Blood Diseases Hospital, Chinese Academy of Medical Sciences and Peking Union Medical College Tianjin China; ^3^ Sir Run Run Shaw Hospital of Zhejiang University School of Medicine Hangzhou Zhejiang China; ^4^ The First Affiliated Hospital of Bengbu Medical University Bengbu Anhui China; ^5^ Gansu Provincial Hospital Lanzhou Gansu China; ^6^ Mundipharma Research Ltd Cambridge UK

**Keywords:** antifungal agents, candidemia, caspofungin, China, echinocandins, invasive candidiasis, Phase 3 trial, rezafungin

## Abstract

**Background:**

The global double‐blind, randomised, Phase 3 ReSTORE trial (NCT03667690) demonstrated noninferiority of rezafungin versus caspofungin for all‐cause mortality at Day 30 and global cure at Day 14 in patients with candidemia and/or invasive candidiasis.

**Objectives:**

We report outcomes for patients from China (ReSTORE China), comprising participants enrolled in the original ReSTORE trial (*n* = 11) and from an extended, China‐only phase (*n* = 47) implemented to fulfill Chinese regulatory requirements.

**Methods:**

Patients with candidemia/invasive candidiasis were randomised 1:1 to intravenous rezafungin (400 mg loading, then 200 mg once weekly) or caspofungin (70 mg loading, then 50 mg once daily) for ≤ 4 weeks. Primary endpoints were all‐cause mortality at Day 30 and global cure at Day 14 in the modified intent‐to‐treat population. Between October 2018 and March 2024, 58 patients were randomised and received study treatment (rezafungin *n* = 28 [modified intent‐to‐treat *n* = 27], caspofungin *n* = 30 [modified intent‐to‐treat *n* = 28]).

**Results:**

All‐cause mortality at Day 30 was 33.3% (9/27) for rezafungin versus 35.7% (10/28) for caspofungin (difference −2.4% [95% confidence interval −27.0–22.6]). Global cure at Day 14 was 48.1% (13/27) versus 46.4% (13/28), respectively (weighted difference 0.3% [95% confidence interval −25.4–26.3]). Day 5 and 14 mycological eradication rates were 70.4% and 63.0% for rezafungin versus 71.4% and 67.9% for caspofungin, respectively. Safety and tolerability profiles were similar between groups.

**Conclusions:**

Rezafungin demonstrated similar efficacy and safety to caspofungin in the ReSTORE China cohort. These findings support the primary ReSTORE analysis and suggest that rezafungin could provide a new treatment option for candidemia/invasive candidiasis in China.

**Trial Registration:**

ClinicalTrials.gov identifier: NCT03667690

## 1 Introduction

It is estimated that, worldwide, there are > 1.5 million cases of candidemia and invasive candidiasis (IC) each year [1]. Most infections occur in hospitals, with an IC incidence of ~100 cases per 100,000 admissions; the highest incidence of IC is observed in intensive care units (ICUs) with 5.5–7.0 cases per 1000 admissions [2]. In China, the incidence of candidemia is reported to be 0.26–0.33 cases per 1000 admissions, and the incidence of IC to be 0.41 cases per 1000 admissions [3, 4, 5]. Furthermore, in ICUs in China, the incidence of IC is reported to be 32 cases per 1000 admissions, with a mortality rate of 37% [6].

*Candida albicans* is the most prevalent *Candida* species (spp.) globally. However, the prevalence of each *Candida* spp. varies geographically, and non‐ *C. albicans* spp. are reportedly becoming more common [7]. Three multicentre surveillance studies of IC in China described the four most prevalent *Candida* spp. to be *C. albicans* , *C. parapsilosis* , *C. tropicalis* and *C. glabrata* [8, 9, 10]. The introduction and transmission of *C. auris* across multiple provinces in China have also recently been reported [11].

Drug resistance, especially multidrug resistance, in *Candida* spp. is a growing problem, particularly in *C. glabrata* and *C. auris* [8, 11, 12, 13]. The increase in fluconazole resistance in *C. parapsilosis* also presents a global health risk [14, 15]. Echinocandins are among the preferred antifungal treatments for candidemia and IC in China and other countries [13, 16, 17]. These antifungals have not only excellent activity against most *Candida* spp., but also good tolerability and few drug–drug interactions [7]. However, most clinical data describing antifungal activity are obtained in Western countries and differences in the geographical prevalence of each *Candida* spp. (with non‐*albicans Candida* spp. being the predominant cause of over two‐thirds of cases of candidemia in China [10]) and their associated susceptibility to common antifungal drugs, including azoles and echinocandins, mean that data obtained in one region of the world may not always be applicable to another.

Rezafungin is a next‐generation echinocandin that has recently been approved for the treatment of adults with candidemia and/or IC in the United States, Europe, Brazil and the UAE [18, 19, 20, 21]. This novel echinocandin has distinctive pharmacokinetic characteristics that differentiate it from other echinocandins. These characteristics include a low clearance rate and an extended half‐life, which enable once‐weekly dosing. Weekly dosing results in high, front‐loaded exposures that may maximise the effect of rezafungin early in the treatment course, therefore increasing its antimicrobial effect [22, 23, 24]. In addition, rezafungin is active against various *Candida* spp., including strains resistant to azoles and some with isolates resistant to the other echinocandins [22, 25, 26].

The efficacy and safety of rezafungin in the treatment of candidemia and IC have been demonstrated in the Phase 2 STRIVE (NCT02734862) and Phase 3 ReSTORE (NCT03667690) trials [27, 28]. In ReSTORE, which was conducted in 15 countries, including China, rezafungin was shown to be noninferior to caspofungin for all‐cause mortality (ACM) at Day 30 and global cure at Day 14 [28]. To fulfill regulatory requirements for drug marketing authorisation in China and allow enrichment of the ReSTORE data with isolates from a region that had limited representation in the original trial, additional patients were recruited to an extended China‐only phase. Data from the ReSTORE China cohort, comprising participants from China who enrolled in the original ReSTORE trial and participants from the extended China‐only phase, are reported herein.

## 2 Patients and Methods

### 2.1 Study Design and Participants

ReSTORE was a global multicentre, double‐blind, randomised, Phase 3, noninferiority trial. Full methodological details have been reported [28]. ReSTORE China comprised participants from China who enrolled in the original ReSTORE trial (*n* = 11) and participants from an extended China‐only phase (*n* = 47).

Eligible patients were adults with systemic signs of candidemia and/or IC plus mycological evidence of infection (based on baseline blood or sterile site specimen cultures collected ≤ 96 h before randomisation) (see the Supporting Information for key eligibility criteria). Patients were randomised in a 1:1 ratio to receive either rezafungin or caspofungin. Rezafungin was administered intravenously (IV) at 400 mg on Day 1 and 200 mg on Day 8. Optional third and fourth doses of rezafungin at 200 mg were administered on Days 15 and 22, respectively, based on the investigator's judgment. Caspofungin was administered IV as a 70 mg loading dose on Day 1, followed by 50 mg/day for ≥ 3 days. After Day 3, patients meeting predefined criteria (e.g., stable clinical status, isolated *Candida* spp. susceptible to fluconazole, all signs and symptoms of candidemia/IC resolved, most recent blood culture negative for *Candida* spp., and no evidence of moderate‐to‐severe hepatic injury (see the Supporting Information) [28]) could switch to oral stepdown therapy (placebo for patients who received rezafungin, fluconazole 200–800 mg/day for patients who received caspofungin). The overall treatment duration was 14–28 days. Assessments are described in the Supporting Information.

### 2.2 Ethics Statement

The authors confirm that the ethical policies of the journal, as noted on the journal's author guidelines page, were adhered to and the appropriate ethical review committee approvals were received. ReSTORE was conducted in accordance with current regulations, the International Conference on Harmonisation Good Clinical Practice guidelines and the Declaration of Helsinki. The protocol was subject to approval by ethics committees or institutional review boards at participating sites. Patients provided written informed consent.

### 2.3 Endpoints

The primary efficacy endpoints were ACM at Day 30 and global cure at Day 14. Secondary efficacy endpoints included global cure at Day 5, and mycological eradication, clinical cure and radiological cure (for patients with IC) at Day 5 and 14. Exploratory efficacy endpoints were the proportion of patients with a negative blood culture at 24 and 48 h post first dose and time to first negative blood culture. These endpoints are defined in the Supporting Information. Additional exploratory endpoints in the China cohort were duration of hospital and intensive care unit (ICU) admissions and readmissions.

Treatment‐emergent adverse events (TEAEs) were recorded and coded using the Medical Dictionary for Regulatory Activities (MedDRA) version 23.0. Safety was also evaluated through physical examination findings, vital signs, laboratory tests and electrocardiogram results.

### 2.4 Statistical Analysis

From a clinical perspective (to assess efficacy and safety in patients from China who may present with distinct pathogenic strains and drug susceptibility) and independent of any statistical considerations, the regulatory authorities in China requested that a certain proportion of the overall ReSTORE study population (from the original ReSTORE trial and the extended China‐only phase) should be from China.

In the China cohort, primary and secondary efficacy endpoints were evaluated in the modified intent‐to‐treat (mITT) population using a two‐sided 95% confidence interval (CI) for the difference between the rezafungin and the caspofungin group (rezafungin – caspofungin), calculated using Miettinen and Nurminen methodology. For endpoints adjusted based on the randomisation strata, Cochran–Mantel–Haenszel weights were applied. Noninferiority was not tested as this is a subgroup of the primary study. Time to negative blood culture was analysed using Kaplan–Meier methods. Descriptive statistics were used to summarise safety endpoints in the safety population.

Study populations are defined in Supporting Information. All analyses were conducted using SAS version 9.4 or higher.

## 3 Results

### 3.1 Patients

**FIGURE 1:**
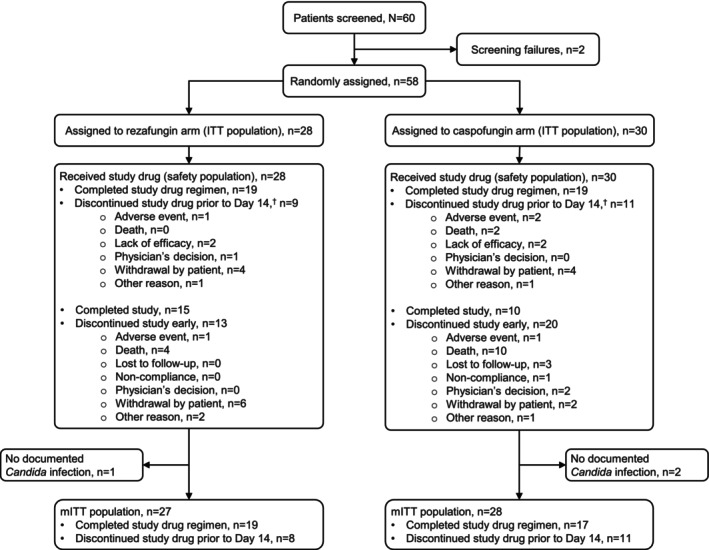
Patient disposition. The disposition and flow of patients in the study are shown, including screening, randomisation, study (drug) discontinuation, study completion and analysis populations. ^†^Patients who discontinued the study drug prior to Day 14 did not have to discontinue the study; they may have continued in the study for the safety analysis. ITT, intent‐to‐treat; mITT, modified intent‐to‐treat.

Between October 12, 2018, and March 31, 2024, 60 patients with candidemia and/or IC from China were screened; 58 patients who met the eligibility criteria for the study were randomised and included in the intent‐to‐treat (ITT) population (rezafungin *n* = 28, caspofungin *n* = 30) (Figure 1). All 58 patients received ≥ 1 dose of the study treatment and were included in the safety population (rezafungin *n* = 28; caspofungin *n* = 30). Fifty‐five patients were included in the mITT population (rezafungin *n* = 27; caspofungin *n* = 28; as defined in the Supporting Information) that was used for the efficacy analyses.

Patient baseline characteristics were similar between the two treatment groups (Table 1). The majority of patients had a modified Acute Physiology and Chronic Health Evaluation (APACHE) II score of < 20 (rezafungin 82.1% [23/28], caspofungin 80.0% [24/30]), an absolute neutrophil count (ANC) of ≥ 500 cells/μL (rezafungin 78.6% [22/28], caspofungin 80.0% [24/30]), and a final diagnosis of candidemia only (rezafungin 92.9% [26/28], caspofungin 86.7% [26/30]). In the mITT population, of patients with candidemia only, 73.1% (19/26) in the rezafungin group and 66.7% (18/27) in the caspofungin group had a catheter present at screening (Table S1).

**TABLE 1:** Patient demographics and baseline characteristics (intent‐to‐treat population).

Characteristic	Treatment group	
	Rezafungin 400/200 mg (*n* = 28)	Caspofungin 70/50 mg (*n* = 30)
Age, *n* (%)		
< 65 years	18 (64.3)	18 (60.0)
≥ 65 years	10 (35.7)	12 (40.0)
Sex, *n* (%)		
Male	22 (78.6)	16 (53.3)
Female	6 (21.4)	14 (46.7)
Race, *n* (%)		
Asian	28 (100.0)	30 (100.0)
Final diagnosis, *n* (%)		
Candidemia only	26 (92.9)	26 (86.7)
IC^a^	2 (7.1)	4 (13.3)
Modified APACHE II score^b^, ^c^, ^d^		
Mean (SD)	14.0 (6.1)	14.4 (7.1)
Score ≥ 20, *n* (%)	4 (14.3)	6 (20.0)
Score < 20, *n* (%)	23 (82.1)	24 (80.0)
Mean body mass index, kg/m^2^ (SD)^c^	21.2 (3.5)	22.2 (4.0)
Absolute neutrophil count, *n* (%)		
< 500 cells/μL	6 (21.4)	6 (20.0)
≥ 500 cells/μL	22 (78.6)	24 (80.0)
Mean estimated creatinine clearance, mL/min (SD)	91.1 (88.1)	75.6 (36.5)
Child–Pugh score, *n* (%)		
< 7	0	0
7–9	1 (3.6)	1 (3.3)
No history of liver disease/not calculated^e^	27 (96.4)	29 (96.7)
Patients with ≥ 1 risk factor for *Candida* in the 3 months prior to enrolment, *n* (%)^f^	28 (100)	30 (100)
Central venous catheter	9 (32.1)	9 (30.0)
Peripherally inserted central catheter	9 (32.1)	11 (36.7)
Active malignancy	6 (21.4)	9 (30.0)
Broad‐spectrum antibiotic therapy	22 (78.6)	19 (63.3)
Diabetes mellitus	3 (10.7)	8 (26.7)
Immunosuppression	8 (28.6)	7 (23.3)
Major surgery	5 (17.9)	6 (20.0)
Total parenteral nutrition	5 (17.9)	9 (30.0)
Transplant recipient	3 (10.7)	0
Trauma	3 (10.7)	1 (3.3)
End‐stage renal disease/dialysis	2 (7.1)	1 (3.3)
Burns	0	1 (3.3)
Pancreatitis	2 (7.1)	2 (6.7)
Other	12 (42.9)	10 (33.3)
In ICU at time of randomisation, *n* (%)	10 (35.7)	13 (43.3)
Currently mechanically ventilated, *n* (%)	6 (21.4)	11 (36.7)
Dialysis within the previous 3 days, *n* (%)	3 (10.7)	2 (6.7)
Pancreatitis within the previous 10 days, *n* (%)	2 (7.1)	2 (6.7)

Abbreviations: APACHE, Acute Physiology and Chronic Health Evaluation; GCS, Glasgow Coma Scale; IC, invasive candidiasis; ICU, intensive care unit; SD, standard deviation.

^a^ Diagnosis of IC based on evaluation of tissue or fluid culture, or radiological assessment.

^b^ Combined APACHE II and GCS score, calculated as APACHE II + (15 minus GCS score).

^c^ Reported for patients with data available.

^d^ Data missing for one patient.

^e^ Patients with no medical history of liver disease were included in this category, regardless of Child–Pugh score.

^f^ Percentages are not mutually exclusive.

The *Candida* spp. present in the blood and sterile site cultures were balanced between the two treatment groups at baseline (Table 2). *C. albicans* and *C. tropicalis* were the most common spp. present. The minimum inhibitory concentration range for rezafungin by baseline *Candida* spp. (analysed *post hoc*) is shown in Table S2.

**TABLE 2:** *Candida* species present in the blood and sterile site cultures at baseline (modified intent‐to‐treat population).

*Candida* spp.^a^	Treatment group, *n* (%)	
	Rezafungin 400/200 mg (*n* = 27)^b^	Caspofungin 70/50 mg (*n* = 28)^b^
*C. albicans*	9 (33.3)	9 (32.1)
*C. tropicalis*	10 (37.0)	8 (28.6)
*C. glabrata*	3 (11.1)	3 (10.7)
*C. parapsilosis*	5 (18.5)	6 (21.4)
*C. guilliermondii*	0	1 (3.6)
*C. guilliermondii* var. *membranifaciens*	0	1 (3.6)

Abbreviation: spp., species.

^a^ Cultures at baseline were collected ≤ 96 h before randomisation or prior to the first dose of the study drug after randomisation.

^b^ No patients in either treatment group had multiple spp.

### 3.2 Treatment Exposure

In the safety population, the median duration of study drug exposure in patients receiving intravenous (IV) therapy only (rezafungin or caspofungin) was 14.0 days in both the rezafungin and the caspofungin group (interquartile range [IQR] 6.0–17.5 and 7.0–15.0, respectively). During the study period, three (10.7%) patients in the rezafungin group and seven (23.3%) in the caspofungin group who met predefined criteria switched to oral stepdown therapy (placebo for patients who received rezafungin, fluconazole for patients who received caspofungin) for a median duration of 10.0 days (IQR 8.0–12.0) and 6.0 days (IQR 6.0–7.0), respectively. The median duration of IV and oral therapy combined was 14.0 days for both rezafungin (IQR 6.0–19.0) and caspofungin (IQR 7.0–21.0).

### 3.3 Primary Endpoints

This study had two primary endpoints: ACM at Day 30 and global cure at Day 14 in the mITT population. At Day 30, 33.3% (9/27) of patients in the rezafungin group and 35.7% (10/28) in the caspofungin group were deceased or had an unknown survival status (treatment difference for ACM at Day 30 –2.4% [95% CI −27.0–22.6]) (Table 3). Global cure at Day 14 was observed in 48.1% (13/27) of patients in the rezafungin group and 46.4% (13/28) in the caspofungin group (weighted treatment difference 0.3% [95% CI −25.4–26.3]) (Table 3).

**TABLE 3:** All‐cause mortality at Day 30 and global response at Day 14 (modified intent‐to‐treat population).

Endpoint	Treatment group, *n* (%)		Treatment difference, % (95% CI)
	Rezafungin 400/200 mg (*n* = 27)	Caspofungin 70/50 mg (*n* = 28)	
All‐cause mortality at Day 30			
Died or unknown survival status^a^	9 (33.3)	10 (35.7)	−2.4 (−27.0–22.6)^b^
Known to have died	5 (18.5)	7 (25.0)	
Unknown survival status	4 (14.8)	3 (10.7)	
Global response at Day 14, assessed by DRC			
Cure	13 (48.1)	13 (46.4)	0.3 (−25.4–26.3)^c^
Failure	11 (40.7)	12 (42.9)	
Indeterminate	3 (11.1)	3 (10.7)	

Abbreviations: ANC, absolute neutrophil count; APACHE, Acute Physiology and Chronic Health Evaluation; CI, confidence interval; DRC, Data Review Committee.

^a^ Patients deceased on or before Day 30, or with an unknown survival status.

^b^ Two‐sided 95% CI for the observed difference (%) (rezafungin − caspofungin group).

^c^ Two‐sided 95% CI for the weighted difference (%) (rezafungin − caspofungin group), adjusted for the two randomisation strata of diagnosis and APACHE II score/ANC.

### 3.4 Secondary and Exploratory Efficacy Endpoints

The secondary efficacy endpoints included global cure at Day 5 and mycological eradication at Day 5 and 14 in the mITT population. Exploratory efficacy endpoints were the proportion of patients with a negative blood culture at 24 and 48 h post first dose, and time to first negative blood culture.

Global cure at Day 5 was seen in 33.3% (9/27) of patients in the rezafungin group and 35.7% (10/28) in the caspofungin group (treatment difference −2.4% [95% CI −27.0–22.6]) (Table 4). Mycological eradication at Day 5 was seen in 70.4% (19/27) of patients in the rezafungin group and 71.4% (20/28) in the caspofungin group. At Day 14, mycological eradication was seen in 63.0% (17/27) and 67.9% (19/28) of patients in the rezafungin group and the caspofungin group, respectively (Table 4).

**TABLE 4:** Secondary and exploratory efficacy endpoints (modified intent‐to‐treat population).

Endpoint	Treatment group		
	Rezafungin 400/200 mg (*n* = 27)	Caspofungin 70/50 mg (*n* = 28)	Treatment difference, % (95% CI)^a^
Day 5 endpoints			
Global cure, assessed by DRC, *n* (%)	9 (33.3)	10 (35.7)	−2.4 (−27.0–22.6)
Mycological eradication,^b^ *n* (%)	19 (70.4)	20 (71.4)	−1.1 (−25.1–22.9)
Investigator assessment of clinical cure, *n* (%)	6 (22.2)	6 (21.4)	0.8 (−21.5–23.3)
Radiological cure, *n*/*N* ^c^	0/0	1/1	—
Day 14 endpoints			
Global cure, assessed by DRC, *n* (%)	13 (48.1)	13 (46.4)	0.3 (−25.4–26.3)^d^
Mycological eradication,^b^ *n* (%)	17 (63.0)	19 (67.9)	−4.9 (−29.5–20.1)
Investigator assessment of clinical cure, *n* (%)	12 (44.4)	15 (53.6)	−9.1 (−34.2–17.2)
Radiological cure, *n*/*N* ^c^	0/1	1/2	−50.0 (−93.1–64.2)
Exploratory endpoint			
Patients with negative blood culture, *n*/*N* (%)			
At 24 h	5/24 (20.8)	4/24 (16.7)	—
At 48 h	14/22 (63.6)	12/23 (52.2)	—

Abbreviations: ANC, absolute neutrophil count; APACHE, Acute Physiology and Chronic Health Evaluation; CI, confidence interval; DRC, Data Review Committee; IC, invasive candidiasis.

^a^ Two‐sided 95% CI for the observed difference (%) (rezafungin – caspofungin group), unless otherwise stated.

^b^ Programmatically derived from the outcome definition; includes both documented and presumed mycological eradication.

^c^ In patients with IC documented by radiological/imaging evidence; note: some patients experienced progression to IC after Day 5.

^d^ Two‐sided 95% CI for the weighted difference (%) (rezafungin − caspofungin group), adjusted for the two randomisation strata of diagnosis and APACHE II score/ANC.

At 24 h, the proportion of patients with a negative blood culture was 20.8% (5/24) in the rezafungin group and 16.7% (4/24) in the caspofungin group. At 48 h, the proportions were 63.6% (14/22) and 52.2% (12/23), respectively (Table 4). The median time to first negative blood culture was 46.4 h (95% CI 32.4–90.3) in the rezafungin group and 48.9 h (95% CI 37.8–94.3) in the caspofungin group.

### 3.5 Exploratory Analyses of Hospital and Intensive Care Unit Admissions

In the mITT population, in patients who remained alive during hospitalisation (*n* = 23 in both treatment groups), the median duration of hospital stay across all admissions was 25.0 days (IQR 16.0–45.0) in the rezafungin group and 21.0 days (IQR 15.0–31.0) in the caspofungin group. The median duration of ICU admission was 10.0 days (IQR 6.0–24.0; *n* = 9) and 10.5 days (IQR 4.0–22.0; *n* = 6) in the rezafungin and the caspofungin group, respectively. Two (7.4%) patients receiving rezafungin and four (14.3%) patients receiving caspofungin were newly admitted to the ICU on or after Day 1 of the study.

None of the patients discharged from hospital before Day 30 was readmitted by Day 30. By follow‐up (Days 52–59), 4.5% (1/22) of patients discharged on or after Day 1 in the rezafungin group and 9.5% (2/21) in the caspofungin group had been readmitted. Of the patients admitted to the ICU during their initial hospitalisation and subsequently discharged from the ICU before Day 30, 10.0% (1/10) in the rezafungin group and 22.2% (2/9) in the caspofungin group were readmitted to the ICU by Day 30. By follow‐up, 9.1% (1/11) of patients in the rezafungin group and 30.0% (3/10) in the caspofungin group were readmitted to the ICU.

In a *post hoc* analysis, study investigators indicated that they would have considered discharging 9.1% (5/55) of patients sooner if once‐weekly IV rezafungin had been available (11.1% [3/27] of patients in the rezafungin group and 7.1% [2/28] in the caspofungin group).

### 3.6 Safety

In the safety population, TEAEs were experienced by 96.4% (27/28) of patients in the rezafungin group and 96.7% (29/30) in the caspofungin group (Table 5). The most common TEAE, experienced by > 20% of patients in both treatment groups, was hypokalaemia. Study drug‐related TEAEs were reported in 25.0% (7/28) and 26.7% (8/30) of patients in the rezafungin and the caspofungin group, respectively; none led to study drug discontinuation. Study drug‐related TEAEs that were reported in > 1 patient were raised alanine aminotransferase (rezafungin 7.1% [2/28], caspofungin 3.3% [1/30]), hypokalaemia (rezafungin 3.6% [1/28], caspofungin 6.7% [2/30]) and diarrhoea (rezafungin 0.0%, caspofungin 6.7% [2/30]) (Table S3). TEAEs led to study drug discontinuation in 3.6% (1/28) and 6.7% (2/30) of patients in the rezafungin and the caspofungin group, respectively. TEAEs that led to study drug discontinuation were respiratory failure (rezafungin group), *Aspergillus* infection (caspofungin group) and pneumonia (caspofungin group).

**TABLE 5:** Treatment‐emergent adverse events in patients with ≥ 1 treatment‐emergent adverse event (safety population).

TEAE	Treatment group, *n* (%)	
	Rezafungin 400/200 mg (*n* = 28)	Caspofungin 70/50 mg (*n* = 30)
Any TEAE	27 (96.4)	29 (96.7)
Study drug‐related TEAEs	7 (25.0)	8 (26.7)
SAE	13 (46.4)	17 (56.7)
Study drug‐related SAE	0	0
SAE leading to death	7 (25.0)	11 (36.7)
TEAE leading to study drug discontinuation	1 (3.6)	2 (6.7)
TEAE leading to study discontinuation	4 (14.3)	4 (13.3)
TEAEs in ≥ 10% of patients in either group		
Hypokalaemia	6 (21.4)	7 (23.3)
Anaemia	5 (17.9)	2 (6.7)
Diarrhoea	4 (14.3)	2 (6.7)
Hyponatraemia	4 (14.3)	1 (3.3)
Pneumonia	4 (14.3)	5 (16.7)
Abdominal pain	3 (10.7)	1 (3.3)
Septic shock	3 (10.7)	4 (13.3)
Weight decreased	3 (10.7)	0
Hypoalbuminaemia	2 (7.1)	4 (13.3)
Platelet count decreased	2 (7.1)	3 (10.0)
Pyrexia	2 (7.1)	3 (10.0)
Respiratory failure	2 (7.1)	3 (10.0)
Hypocalcaemia	1 (3.6)	5 (16.7)
Hypoproteinaemia	1 (3.6)	4 (13.3)

Abbreviations: SAE, serious adverse event; TEAE, treatment‐emergent adverse event.

Serious adverse events (SAEs) were experienced by 46.4% (13/28) of patients in the rezafungin group and 56.7% (17/30) in the caspofungin group. In the rezafungin group, SAEs that were reported in > 1 patient were gastrointestinal haemorrhage (7.1% [2/28]), multiple organ dysfunction syndrome (7.1% [2/28]) and sepsis (7.1% [2/28]). In the caspofungin group, SAEs that were reported in > 1 patient were septic shock (13.3% [4/30]) and pneumonia (6.7% [2/30]). No study drug‐related SAEs were reported.

## 4 Discussion

Although formal inferential statistical analysis was not undertaken, data from the ReSTORE China cohort (herein referred to as the China cohort) of patients with candidemia and IC suggested that rezafungin and caspofungin have similar efficacy with respect to the primary (ACM at Day 30 and global cure at Day 14), secondary (including global cure at Day 5 and mycological eradication at Days 5 and 14) and exploratory endpoints (including time to negative blood culture, duration of hospital/ICU admissions and readmissions). Rezafungin had a comparable safety and tolerability profile to caspofungin. For both rezafungin and caspofungin, the nature, intensity and seriousness of TEAEs were consistent with those reported in the primary analysis of the ReSTORE trial and the Phase 2 STRIVE trial [27, 28], as well as their established safety profiles [18, 19, 29].

In the China cohort, both ACM at Day 30 and global cure at Day 14 were comparable between the treatment groups; this is in accordance with the findings of the primary ReSTORE analysis (herein referred to as the primary analysis), in which rezafungin was shown to be noninferior to caspofungin for these primary endpoints [28]. For ACM at Day 30, the treatment difference (rezafungin − caspofungin) was 2.4% (95% CI −9.7–14.4) in the primary analysis [28] and −2.4% (95% CI −27.0–22.6) in the China cohort. For global cure at Day 14, the weighted treatment difference was −1.1% (95% CI −14.9–12.7) in the primary analysis and 0.3% (95% CI −25.4–26.3) in the China cohort.

Despite the similarity between the two treatment groups in the primary endpoints, higher overall Day 30 ACM rates and lower Day 14 global cure rates were observed in the China cohort than in the primary analysis [28]. We believe that several factors may have contributed to this finding. A higher proportion of patients in the China cohort (14.8% and 10.7% of patients in the rezafungin and caspofungin groups, respectively) than in the primary analysis (3.2% of patients in each group) had unknown survival status at Day 30. Given that patients with unknown survival status were considered deceased for the purposes of calculating ACM, the higher incidence in the China cohort is likely to have inflated the mortality rate; this is probably compounded by the smaller sample size. The higher Day 30 ACM rates and lower Day 14 global cure rates in the China cohort may also be related to the observed ANC levels and APACHE II scores, with both being indicators of more severe disease. A higher proportion of patients in the China cohort than in the primary analysis had ANC levels of < 500 cells/μL (respective proportions were 21.4% vs. 9.0% for rezafungin and 20.0% vs. 6.1% for caspofungin) and a higher mean modified APACHE II score (respective mean scores were 14.0 vs. 12.5 for rezafungin and 14.4 vs. 13.1 for caspofungin) [28]. In addition, the data may have been affected by the different *Candida* spp. present in the two study populations (and consequently different echinocandin susceptibility profiles), which could reflect geographical variations in prevalence [7, 8, 9, 11].

Results from the primary analysis suggested that there were potential early treatment benefits for rezafungin versus caspofungin. This was indicated by a shorter median time to first negative blood culture (23.9 h vs. 27.0 h, respectively), higher proportion of patients with negative blood culture at 24 h (53.7% vs. 46.2%, respectively) and 48 h (74.2% vs. 64.1%, respectively), and higher rate of mycological eradication at Day 5 (treatment difference 7.1% [95% CI −6.6–20.6]) in the rezafungin group than the caspofungin group [28]. The median time to first negative blood culture was numerically longer in the China cohort (rezafungin 46.4 h, caspofungin 48.9 h) than in the primary analysis, with a less pronounced difference between the two treatment groups. There was also little difference between the treatment groups in the rate of mycological eradication at Day 5 (−1.1% [95% CI −25.1–22.9]) in the China cohort; however, rates in both groups were similar to those in the primary analysis. Possible explanations for the differences between the China cohort and the primary analysis could include the smaller sample size in the China cohort (making it more difficult to accurately detect differences), differences in baseline characteristics (e.g., ethnicity, infection type, ANC levels, *Candida* risk factors and catheter placement/removal), and the distribution of *Candida* spp. between the two populations.

A population pharmacokinetic model has been reported for rezafungin, which was developed using data from healthy participants, hepatically impaired patients and patients with candidemia and/or IC [30]. Covariate analysis did not identify any patient factors that were associated with clinically meaningful changes in rezafungin pharmacokinetics, indicating that a common dose regimen is adequate for all adult patients. An update to the population pharmacokinetic model to include pharmacokinetic data from patients in the ReSTORE China extension study enabled assessment of rezafungin exposure in patients from China and comparison with the non‐China cohort [31]. The analysis showed no clinically meaningful differences in rezafungin exposure parameters between patients from China and patients from elsewhere, supporting a lack of requirement for dose adjustments in patients from China [31].

Given the geographical variation in the prevalence and patterns of *Candida* spp. and antifungal resistance [7], extension cohorts, such as this one, are important because they provide region‐specific data that can be used to tailor treatment strategies to the local clinical landscape, in this case, in China. Although the small sample in the China cohort prohibited formal statistical analysis, the efficacy and safety findings were consistent with those for the main primary analysis, providing confidence in the findings. No separate hypothesis was formulated for the extension cohort, which was conducted specifically to meet regulatory requirements for evaluating efficacy and safety in a sample of patients from China. In the future, real‐world post‐marketing studies will further evaluate the effectiveness and safety of rezafungin in patients from China, while antimicrobial susceptibility testing results of isolates will be monitored to enrich the dataset and capture regional variations in pathogen distribution and antifungal resistance. In addition to the small sample size, limitations reported for the original ReSTORE trial apply here also, such as the exclusion of children and patients with specific forms of IC [28].

In this ReSTORE China cohort, rezafungin demonstrated comparable efficacy and safety to caspofungin in patients with candidemia or IC. These findings support the primary analysis of the Phase 3 ReSTORE trial and suggest that rezafungin could provide a new treatment option for candidemia and/or IC in China.

## Author Contributions

**Haihui Huang:** data curation, investigation, project administration, resources, supervision, validation, writing – review and editing. **Sizhou Feng:** data curation, investigation, project administration, resources, writing – review and editing. **Yunsong Yu:** data curation, investigation, project administration, resources, writing – review and editing. **Yong Zhang:** data curation, investigation, project administration, resources, writing – review and editing. **Yuan Yuan:** data curation, investigation, project administration, resources, writing – review and editing. **Laura Cox:** data curation, investigation, project administration, resources, writing – review and editing. **Yingyuan Zhang:** conceptualisation, data curation, investigation, methodology, project administration, resources, supervision, validation, writing – review and editing.

## Conflicts of Interest

Yingyuan Zhang, Haihui Huang, Sizhou Feng, Yunsong Yu, Yong Zhang and Yuan Yuan report no conflicts of interest. Laura Cox is an employee of Mundipharma Research Ltd.

## Supporting information


**Data S1:** myc70122‐sup‐0001‐DataS1.pdf.

## Data Availability

Access to the respective study protocols and anonymised data can be requested by contacting enquiries@napp.co.uk. Each request will be reviewed by the sponsor for scientific merit.

## References

[1] D. W. Denning , “Global Incidence and Mortality of Severe Fungal Disease,” Lancet Infectious Diseases 24 (2024): e428–e438, 10.1016/s1473-3099(23)00692-8. 38224705

[2] C. Lass‐Flörl , S. S. Kanj , N. P. Govender , G. R. Thompson , L. Ostrosky‐Zeichner , and M. A. Govrins , “Invasive Candidiasis,” Nature Reviews Disease Primers 10 (2024): 20, 10.1038/s41572-024-00503-3. 38514673

[3] Z. Zeng , Y. Ding , G. Tian , et al., “A Seven‐Year Surveillance Study of the Epidemiology, Antifungal Susceptibility, Risk Factors and Mortality of Candidaemia Among Paediatric and Adult Inpatients in a Tertiary Teaching Hospital in China,” Antimicrobial Resistance and Infection Control 9 (2020): 133, 10.1186/s13756-020-00798-3. 32799915 PMC7429891

[4] L. Chen , Z. Xie , and J. Jian , “Epidemiology and Risk Factors of Candidemia a 8‐Year Retrospective Study From a Teaching Hospital in China,” Infection and Drug Resistance 17 (2024): 3415–3423, 10.2147/idr.S471171. 39131515 PMC11317046

[5] Z. R. Zeng , G. Tian , Y. H. Ding , K. Yang , J. B. Liu , and J. Deng , “Surveillance Study of the Prevalence, Species Distribution, Antifungal Susceptibility, Risk Factors and Mortality of Invasive Candidiasis in a Tertiary Teaching Hospital in Southwest China,” BMC Infectious Diseases 19 (2019): 939, 10.1186/s12879-019-4588-9. 31699043 PMC6836498

[6] F. Guo , Y. Yang , Y. Kang , et al., “Invasive Candidiasis in Intensive Care Units in China: A Multicentre Prospective Observational Study,” Journal of Antimicrobial Chemotherapy 68 (2013): 1660–1668, 10.1093/jac/dkt083. 23543609

[7] P. G. Pappas , M. S. Lionakis , M. C. Arendrup , L. Ostrosky‐Zeichner , and B. J. Kullberg , “Invasive Candidiasis,” Nature Reviews Disease Primers 4 (2018): 18026, 10.1038/nrdp.2018.26. 29749387

[8] M. Xiao , Z. Y. Sun , M. Kang , et al., “Five‐Year National Surveillance of Invasive Candidiasis: Species Distribution and Azole Susceptibility From the China Hospital Invasive Fungal Surveillance Net (CHIF‐NET) Study,” Journal of Clinical Microbiology 56 (2018): e00577−18, 10.1128/jcm.00577-18. 29743305 PMC6018329

[9] L. N. Guo , S. Y. Yu , M. Xiao , et al., “Species Distribution and Antifungal Susceptibility of Invasive Candidiasis: A 2016–2017 Multicenter Surveillance Study in Beijing, China,” Infection and Drug Resistance 13 (2020): 2443–2452, 10.2147/idr.S255843. 32765018 PMC7381087

[10] M. Xiao , S. C. Chen , F. Kong , et al., “Distribution and Antifungal Susceptibility of Candida Species Causing Candidemia in China: An Update From the CHIF‐NET Study,” Journal of Infectious Diseases 221 (2020): S139–S147, 10.1093/infdis/jiz573. 32176789

[11] J. Bing , H. Du , P. Guo , et al., “ *Candida auris*‐Associated Hospitalizations and Outbreaks, China, 2018–2023,” Emerging Microbes & Infections 13 (2024): 2302843, 10.1080/22221751.2024.2302843. 38238874 PMC10802803

[12] N. Ye , Z. Liu , W. Tang , X. Li , W. Chu , and Q. Zhou , “Systematic Characterization of Epidemiology, Antifungal Susceptibility, Risk Factors and Outcomes of Candidaemia: A Six‐Year Chinese Study,” Infection and Drug Resistance 15 (2022): 4887–4898, 10.2147/idr.S378629. 36051656 PMC9426866

[13] Chinese Adult Candidiasis Diagnosis and Management Expert Consensus Group , “Chinese Consensus on the Diagnosis and Management of Adult Candidiasis” [in Chinese], Zhonghua Nei Ke Za Zhi 59 (2020): 5–17. 31887830 10.3760/cma.j.issn.0578-1426.2020.01.002

[14] O. A. Cornely , R. Sprute , M. Bassetti , et al., “Global Guideline for the Diagnosis and Management of Candidiasis: An Initiative of the ECMM in Cooperation With ISHAM and ASM,” Lancet Infectious Diseases 25 (2025): e280–e293, 10.1016/S1473-3099(24)00749-7. 39956121

[15] P. Escribano and J. Guinea , “Fluconazole‐Resistant *Candida parapsilosis*: A New Emerging Threat in the Fungi Arena,” Frontiers in Fungal Biology 3 (2022): 1010782, 10.3389/ffunb.2022.1010782. 37746202 PMC10512360

[16] O. A. Cornely , M. Bassetti , T. Calandra , et al., “ESCMID* Guideline for the Diagnosis and Management of Candida Diseases 2012: Non‐Neutropenic Adult Patients,” Clinical Microbiology and Infection 18 (2012): 19–37, 10.1111/1469-0691.12039. 23137135

[17] P. G. Pappas , C. A. Kauffman , D. R. Andes , et al., “Clinical Practice Guideline for the Management of Candidiasis: 2016 Update by the Infectious Diseases Society of America,” Clinical Infectious Diseases 62 (2016): e1−e50, 10.1093/cid/civ933. 26679628 PMC4725385

[18] Melinta Therapeutics LLC , “REZZAYO^TM^ (Rezafungin for Injection), for Intravenous Use,” US Prescribing Information, 2023, https://www.accessdata.fda.gov/drugsatfda_docs/label/2023/217417s000lbl.pdf.

[19] Mundipharma GmbH , “REZZAYO 200 mg Powder for Concentrate for Solution for Infusion. Summary of Product Characteristics,” 2025, https://www.ema.europa.eu/en/documents/product‐information/rezzayo‐epar‐product‐information_en.pdf.

[20] Mundipharma , “Mundipharma Acquires All Assets and Rights Related to REZZAYO® (Rezafungin), Reinforcing Continued Commitment to Management of Infectious Diseases and Specialty Care Therapeutic Area,” 2024, https://www.mundipharma.com/mundipharma‐acquires‐all‐assets‐and‐rights‐related‐to‐rezzayo.

[21] Mundipharma Brasil Produtos Médicos e Farmacêuticos Ltda , “REZZAYO® (Rezafungina) Bula do Profissional de Saúde [Prescribing Information (Brazil)],” 2025, https://br.mundipharma.com/sites/mundi‐pharma‐brazil/files/2025‐01/Rezzayo%20Bula%20Profissional.pdf.

[22] Y. Y. Ham , J. S. Lewis, 2nd , and G. R. Thompson, 3rd , “Rezafungin: A Novel Antifungal for the Treatment of Invasive Candidiasis,” Future Microbiology 16 (2021): 27–36, 10.2217/fmb-2020-0217. 33438477

[23] V. Ong , K. D. James , S. Smith , and B. R. Krishnan , “Pharmacokinetics of the Novel Echinocandin CD101 in Multiple Animal Species,” Antimicrobial Agents and Chemotherapy 61 (2017): e01626−16, 10.1128/AAC.01626-16. 28137817 PMC5365648

[24] D. Andes , R. J. Bruggemann , S. Flanagan , et al., “The Distinctive Pharmacokinetic Profile of Rezafungin, a Long‐Acting Echinocandin Developed in the Era of Modern Pharmacometrics,” Journal of Antimicrobial Chemotherapy 80 (2025): 18–28, 10.1093/jac/dkae415. 39540899 PMC11695911

[25] A. J. Lepak , M. Zhao , and D. R. Andes , “Pharmacodynamic Evaluation of Rezafungin (CD101) Against *Candida auris* in the Neutropenic Mouse Invasive Candidiasis Model,” Antimicrobial Agents and Chemotherapy 62 (2018): e01572–18, 10.1128/AAC.01572-18. 30181375 PMC6201117

[26] A. J. Lepak , M. Zhao , B. VanScoy , P. G. Ambrose , and D. R. Andes , “Pharmacodynamics of a Long‐Acting Echinocandin, CD101, in a Neutropenic Invasive‐Candidiasis Murine Model Using an Extended‐Interval Dosing Design,” Antimicrobial Agents and Chemotherapy 62 (2018): e02154−17, 10.1128/AAC.02154-17. 29203480 PMC5786781

[27] G. R. Thompson , A. Soriano , A. Skoutelis , et al., “Rezafungin Versus Caspofungin in a Phase 2, Randomized, Double‐Blind Study for the Treatment of Candidemia and Invasive Candidiasis: The STRIVE Trial,” Clinical Infectious Diseases 73 (2021): e3647–e3655, 10.1093/cid/ciaa1380. 32955088 PMC8662762

[28] G. R. Thompson, 3rd , A. Soriano , O. A. Cornely , et al., “Rezafungin Versus Caspofungin for Treatment of Candidaemia and Invasive Candidiasis (ReSTORE): A Multicentre, Double‐Blind, Double‐Dummy, Randomised Phase 3 Trial,” Lancet 401 (2023): 49–59, 10.1016/s0140-6736(22)02324-8. 36442484

[29] Fresenius Kabi , “Caspofungin Acetate for Injection, for Intravenous Use,” US Prescribing Information, 2021, https://www.accessdata.fda.gov/drugsatfda_docs/label/2021/206110s003lbl.pdf.

[30] S. Roepcke , J. Passarell , H. Walker , and S. Flanagan , “Population Pharmacokinetic Modeling and Target Attainment Analyses of Rezafungin for the Treatment of Candidemia and Invasive Candidiasis,” Antimicrobial Agents and Chemotherapy 67 (2023): e00916−23, 10.1128/aac.00916-23. 38014945 PMC10720538

[31] G. R. Thompson , H. Huang , S. Feng , et al., “Rezafungin Versus Caspofungin for the Treatment of Candidemia and Invasive Candidiasis: Results From the Double‐Blind, Randomized Phase 3 ReSTORE Trial Including the China Extension Study,” Open Forum Infectious Diseases 12 (2025): ofaf555, 10.1093/ofid/ofaf555. PMC12461853 41018703

