# Supplementary material for: Efficacy and Safety of Rezafungin Versus Caspofungin for the Treatment of Candidemia and Invasive Candidiasis in a China Cohort of a Double‐Blind, Randomised, Phase 3 Trial (ReSTORE China)
Source: Mycoses. 2025 Nov 5;68(11):e70122. doi: 10.1111/myc.70122 (PMC12589930; doi:10.1111/myc.70122)
Supplement: Supplementary file 1 — Data S1: myc70122‐sup‐0001‐DataS1.pdf. [file MYC-68-e70122-s001.pdf]

SUPPLEMENTAL MATERIAL

Supplemental methods

Key eligibility criteria

Patients included in the study were adults aged ≥ 18 years; had an established mycological diagnosis of candidemia and/or invasive candidiasis (IC) from a blood or normally sterile site sample ≤ 96 h before randomisation; had ≥ 1 systemic sign of infection attributable to candidemia or IC (e.g., fever, hypothermia, hypotension, tachycardia, tachypnoea or local signs of inflammation) appearing from 12 h before the qualifying positive culture to the time of randomisation; and were willing to initiate or continue medical treatment to cure infections.

Any of the following resulted in exclusion: presence of septic arthritis in a prosthetic joint; osteomyelitis; endocarditis or myocarditis; meningitis, endophthalmitis, chorioretinitis or any central nervous system infection; chronic disseminated candidiasis; urinary tract candidiasis due to ascending *Candida* infection secondary to obstruction or surgical instrumentation of the urinary tract; receipt of a systemic antifungal agent at an approved dose to treat candidemia for > 48 h (e.g., > 2 doses of a once-daily agent or > 4 doses of a twice-daily agent) in the 96 h before randomisation; alanine aminotransferase or aspartate aminotransferase levels > 10 × the upper limit of normal; severe hepatic impairment with history of chronic cirrhosis (Child–Pugh score of > 9); presence of an indwelling catheter or device that could not be removed or an abscess that could not be drained and was likely to be the source of candidemia or IC; or known hypersensitivity to echinocandins.

Criteria for oral stepdown therapy

An oral stepdown therapy was allowed in both treatment groups provided the following criteria were met:

- Able to take oral medication
- Had received ≥ 3 days of intravenous study drug (or the minimum duration of intravenous therapy advised by the site's national/regional/local guidelines, whichever was greater)

- *Candida* spp. isolated was susceptible to fluconazole
- All signs and symptoms of candidemia and/or invasive candidiasis that were present at baseline had resolved
- Clinical status was considered stable based on investigator assessment
- Most recent blood culture was drawn following the first dose of study drug AND ≥ 48 h prior to oral study drug initiation AND was negative for *Candida* spp.
- No evidence of moderate or severe hepatic injury (alanine aminotransferase or aspartate aminotransferase > 5 × the upper limit of normal)
- No history of hypersensitivity to any azole or any other contraindications to the use of fluconazole or its excipients, including concomitant use of the following medications: terfenadine, cisapride, astemizole, erythromycin, pimozide and quinidine
- No personal or family history of long QT interval on electrocardiogram (ECG) syndrome or a prolonged QT interval corrected for heart rate using Fridericia's formula (QTcF) (> 470 msec in males and > 480 msec in females)
- In the investigator's opinion, the patient could tolerate oral fluconazole therapy (by referring to current fluconazole prescribing information)
- Patient's weight was ≤ 130 kg.

Assessments

Clinical symptoms and physical findings were assessed at screening, on Days 5, 14 and 28–30, and at the end of treatment and during follow-up (Days 52–59). Blood cultures were taken daily or every other day until the first negative result, with no subsequent positive result.

For the ReSTORE China cohort, identification of fungal species (spp.) and evaluation of antifungal susceptibility were carried out at a central laboratory in Shanghai, China. Spp. identification was conducted using the VITEK2 COMPACT system (bioMérieux, Marcy-l'Étoile, France). Antifungal susceptibility testing was carried out using Clinical and Laboratory Standards Institute broth microdilution (M27 Ed4) methods, in accordance with quality control guidelines (M27M44S Ed3).

Endpoint definitions

All-cause mortality at Day 30 was defined as the proportion of patients who died on or before Day 30 or patients with an unknown survival status; patients who were alive on Days 28 or 29 but had an unknown survival status at Day 30 were considered to be alive.

Global cure, assessed at Day 5 and Day 14 (and [not reported herein] Day 30, end of treatment and follow-up [Days 52–59]), was defined as clinical cure (as assessed by the investigator), radiological cure (for patients with IC documented by radiological or imaging evidence at baseline) and mycological eradication, as confirmed for all three parameters by an independent blinded Data Review Committee (DRC).

Clinical cure, as assessed by the investigator, was defined as the resolution of all attributable systemic signs and symptoms of candidemia and/or IC that were present at baseline, with no new systemic signs or symptoms attributable to the infection. The definition of clinical cure also required patients to be alive and have had no change in antifungal therapy for the treatment of candidemia and/or IC.

Radiological cure (for patients with IC documented by radiological or imaging evidence at baseline) was defined as an improvement or resolution of the radiological/imaging findings of IC that were present at baseline, with no new radiological/imaging findings attributable to the infection. The definition of radiological cure also required patients to be alive.

Mycological eradication was defined, for patients with a positive blood culture at baseline, as a negative blood culture after the first dose of study drug with no subsequent positive culture. For patients with a positive baseline culture from a normally sterile site (other than blood), mycological eradication was either documented (defined as a negative culture from all normally sterile baseline sites on or prior to the day of assessment) or presumed (by successful clinical outcome [as assessed by the investigator] and radiological outcome [for those with evidence of disease on imaging at baseline], or if a specimen from all normally sterile sites of baseline infection was not available). The definition of mycological eradication

required patients not to have been lost to follow-up on the day of assessment and to have had no change in antifungal therapy for the treatment of candidemia and/or IC.

Time to negative blood culture was calculated as the time from first dose of study drug (in hours) to the time of first negative culture without subsequent positive cultures. For patients with negative blood culture prior to the first dose of study drug without subsequent positive culture, the time to the first negative blood culture was set to zero. Patients were censored if they received an alternative antifungal (i.e., other than study drug) for the treatment of the candidemia, died or were lost to follow-up prior to having the negative blood culture.

Study populations

Demographics and baseline characteristics were summarised in the intent-to-treat (ITT) population, defined as all randomly assigned patients. Catheter placement and efficacy analyses were presented in the modified ITT (mITT) population, defined as all patients in the ITT population with a documented *Candida* infection, confirmed by the central laboratory, who received ≥ 1 dose of the study drug. Study drug exposure and safety analyses were summarised for the safety population, defined as all patients who had received ≥ 1 dose of the study drug.

Supplemental tables

TABLE S1. Catheter type present at screening in patients with candidemia only (modified intent-to-treat population).

	Treatment group, n (%)	
Catheter type	Rezafungin 400/200 mg (*n* = 26)	Caspofungin 70/50 mg (*n* = 27)
Any catheter	19/26 (73.1)	18/27 (66.7)
Non-tunnelled CVC[a]	5/19 (26.3)	4/18 (22.2)
Tunnelled CVC[a]	4/19 (21.1)	5/18 (27.8)
Implantable port[a]	3/19 (15.8)	1/18 (5.6)
PICC[a]	9/19 (47.4)	10/18 (55.6)
Arterial line[a]	2/19 (10.5)	5/18 (27.8)
Catheter removed within 48 h of diagnosis[a]	4/19 (21.1)	1/18 (5.6)

[a]Denominator is the number of patients with a catheter placement in the mITT population in each treatment group (rezafungin n = 19, caspofungin n = 18).

CVC, central venous catheter; mITT, modified intent-to-treat; PICC, peripherally inserted central catheter.

TABLE S2. Minimum inhibitory concentration range for rezafungin according to baseline *Candida* species (modified intent-to-treat population).

Candida spp.	*n*[a]	MIC range (mg/L)[b]
C. albicans	16	0.002–0.008
C. tropicalis	17	0.016–0.060
C. glabrata	6	0.015–0.030
C. parapsilosis	11	0.500–2.000
C. guilliermondii	1	1.000
C. guilliermondii var. membranifaciens	1	1.000

[a]Number of patients in the mITT population with baseline spp. and susceptibility data available.

[b]For patients with multiple specimen samples with the same baseline spp., the specimen sample with the highest MIC to rezafungin is reported.

MIC, minimum inhibitory concentration; mITT, modified intent-to-treat; spp., species.

TABLE S3. Study drug-related treatment-emergent adverse events by preferred term in patients with ≥ 1 treatment-emergent adverse event (safety population).

	Treatment group, n (%)	
Treatment-emergent adverse event	Rezafungin 400/200 mg (*n* = 28)	Caspofungin 70/50 mg (*n* = 30)
Alanine aminotransferase increased	2 (7.1)	1 (3.3)
Aspartate aminotransferase increased	1 (3.6)	1 (3.3)
Dizziness	1 (3.6)	0
Eosinophil count increased	1 (3.6)	0
Eye pain	1 (3.6)	0
Fatigue	1 (3.6)	0
Hyperkalaemia	1 (3.6)	0
Hypokalaemia	1 (3.6)	2 (6.7)
Hypoproteinaemia	1 (3.6)	0
Infection	1 (3.6)	0
Peripheral neuropathy	1 (3.6)	0
Pneumonia	1 (3.6)	1 (3.3)
Pyrexia	1 (3.6)	1 (3.3)
Acute left ventricular failure	0	1 (3.3)
Blood alkaline phosphatase increased	0	1 (3.3)
Blood bilirubin increased	0	1 (3.3)
Conjunctival haemorrhage	0	1 (3.3)
Constipation	0	1 (3.3)
Cough	0	1 (3.3)
Decreased appetite	0	1 (3.3)
Diarrhoea	0	2 (6.7)
Gamma-glutamyl transferase increased	0	1 (3.3)
Haematochezia	0	1 (3.3)
Haematuria	0	1 (3.3)
Hypoalbuminaemia	0	1 (3.3)
Hypocalcaemia	0	1 (3.3)
Liver injury	0	1 (3.3)

	Treatment group, n (%)	
	Rezafungin	Caspofungin
	400/200 mg	70/50 mg
Treatment-emergent adverse event	(*n* = 28)	(*n* = 30)
Peripheral oedema	0	1 (3.3)
Oxygen saturation decreased	0	1 (3.3)
Proteinuria	0	1 (3.3)
Stomatitis	0	1 (3.3)
Urinary retention	0	1 (3.3)
Urinary tract infection	0	1 (3.3)
Vomiting	0	1 (3.3)
